# Supplementary material for: Microscopic origins of conductivity in molten salts unraveled by computer simulations
Source: Commun Chem. 2021 Jan 27;4:9. doi: 10.1038/s42004-020-00446-2 (PMC9814786; doi:10.1038/s42004-020-00446-2)
Supplement: Supplementary file 1 — Supplemental Information [file 42004_2020_446_MOESM1_ESM.pdf]

# Microscopic origins of conductivity in molten salts unraveled by computer simulations - Supplementary Information

Marie-Madeleine Walz and David van der Spoel*

*Department of Cell and Molecular Biology, Uppsala University, Husargatan 3, Box 596, SE-75124 Uppsala, Sweden*

E-mail: david.vanderspoel@icm.uu.se

Phone: +46 (0) 18 4714205

## Supplementary Methods

### Equations for conductivity evaluation methods

The conductivity of the molten salts were determined using different methods, i.e. the Nernst-Einstein (NE), Green-Kubo (GK), Einstein-Helfand (EH) and the "electric field" (EF) method. For the latter method, we applied an external electric field on the simulation boxes, determined the ion mobility that were used to calculate the conductivity. The NE method neglects ion cross-correlation effects and is supposed to give an upper-bound estimate of the conductivity. The GK and the EH method are supposed to give accurate conductivity values. We find a good agreement between the EF method and the accurate GK and EH method.

**Nernst-Einstein**

The conductivity $\sigma_{\mathrm{NE}}$ is calculated using the NE relation (Supplementary Eqn. 1) that is based on the ion self-diffusion coefficients (e.g. ref.[1–3]):

$$\sigma_{\mathrm{NE}} = \frac{e^2}{k_{\mathrm{B}}T} \sum_i \rho_i z_i^2 D_i \tag{1}$$

where $e$ is the electron charge, $k_{\mathrm{B}}$ is the Boltzmann's constant, $T$ is the temperature, $\rho_i$ is the number density of the respective ion with $N_i/V_{\mathrm{tot}}$, $q_i$ $(= z_i e)$ is the charge and $D_i$ is the self-diffusion coefficient for each component $i$ of the system. The self-diffusion coefficient can be either determined from the mean square displacement[4] or using the Green-Kubo relation that uses the velocity autocorrelation function (see below).

**Green-Kubo**

The Green-Kubo relation for the calculation of the conductivity relies on the electric-current auto correlation function, and is calculated as:[5,6]

$$\sigma_{\mathrm{GK}} = \frac{1}{3Vk_{\mathrm{B}}T} \int_{t=0}^{t=\infty} \langle j(0) \cdot j(t) \rangle dt, \tag{2}$$

where $j(t) = \sum_{i=1}^{N} q_i \cdot v_i(t)$ is the current, with $q_i$ being the ion's charge and $v_i$ its velocity at the time $t$.

**Einstein-Helfand**

The Einstein-Helfand conductivity is determined from the slope of the mean square displacement of the translational dipole moment $M_J = \sum_{i=1}^{N} q_i\ r_i$ with $q_i$ being the charge and $r_i$ being the position of the ion.[7]

$$\lim_{t\to\infty} \langle [M_J(t) - M_J(0)]^2 \rangle = \lim_{t\to\infty} \langle \Delta M_J \rangle = 6Vk_{\mathrm{B}}T\sigma_{\mathrm{EH}}t \tag{3}$$

## Electric field

The electric field conductivity is calculated from the mobility $b$ that has been determined by applying an external electric field and evaluating the drift velocity $v_\mathrm{d}$ in the electric field direction with $b_\mathrm{EF} = v_\mathrm{d}/E$ (see Supplementary Fig. 1) using

$$\sigma_\mathrm{EF} = \sum_i \rho_{N,i}(z_i e) b_i \tag{4}$$

with $\rho_{N,i}$ being the number density, ($z_i$ $e$) being the charge, and $b_i$ the electric mobility.

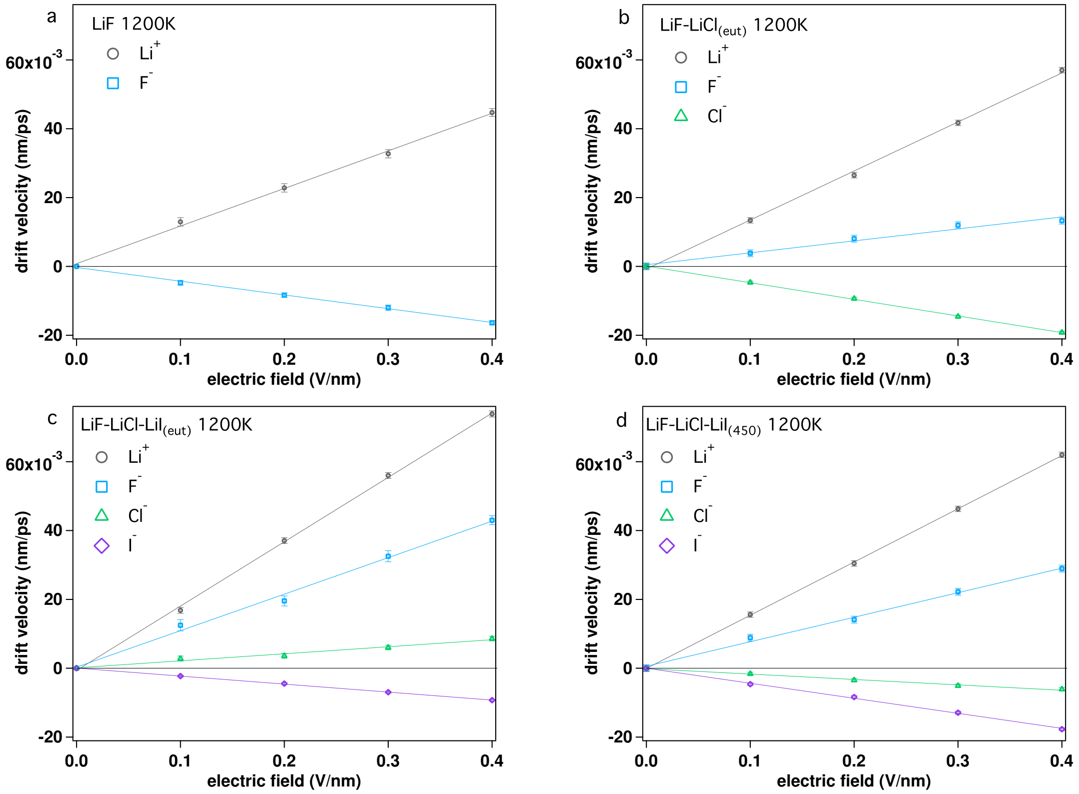


Supplementary Figure 1: Drift velocity of the ions in LiF (a), LiF-LiCl$_{eut}$ (b), LiF-LiCl-LiI$_{eut}$ (c) and LiF-LiCl-LiI$_{450}$ (d) at 1200 K vs. the applied electric field. The slope is equal to the ions mobility.

## Equations for the diffusion coefficient evaluation methods

The diffusion coefficients were evaluated using the Einstein (referred to as msd) and the Green-Kubo (referred to as vac) relation.

### Einstein

The diffusion coefficient is calculated from a linear fit to the mean square displacement (msd) of the ions position $r_i$

$$D_i = \frac{1}{6t} \lim_{t \to \infty} \langle |r_i\,(t) - r_i\,(0)\,|^2 \rangle. \tag{5}$$

### Green-Kubo

The integral of the velocity autocorrelation (vac) function reveals the diffusion coefficient using the Green-Kubo relation:

$$D_i = \frac{1}{3} \int_{t=0}^{t=\infty} \langle v_i(0) \cdot v_i(t) \rangle dt. \tag{6}$$

# Supplementary Note 1

## Tables summarizing the evaluated diffusion coefficients and conductivities

In Supplementary Table 1 and Supplementary Table 2 we list the diffusion coefficients that were evaluated using the msd and vac method (see above). The two different methods show a reasonable agreement, within the estimated average error (approx. 5 %). Furthermore, the density is listed and compared to available experimental data from literature. The agreement is in general good, with the largest deviation for LiF that has a density that is too high. LiCl shows an almost perfect agreement and LiI underestimates the experimental density slightly; the agreement for LiF-LiCl-$LiI_{eut}$ at 773 K is satisfying. Equilibrating the simulation boxes via an NPT simulation and using those densities in NVT simulations instead of experimental densities is recommended; the pressure in the simulation boxes is equilibrated, thus repulsive forces between the ions or the formation of

voids is avoided. Furthermore, the NE, GK and EH conductivities are listed (EF conductivity is listed in Supplementary Table 3), and compared to available experimental conductivity data. The error for the conductivity is estimated to be around 5 %. The agreement is overall satisfactory, in particular for the binary and ternary mixtures. Lastly, the Haven ratio and the NE deviation parameter is listed, showing the deviation of the ideal NE conductivity from the real conductivity. Smaller Haven ratios are observed for LiF and LiCl at 1200 K and for the investigated ternary mixtures at 773 K. For all other compounds at 1200 K the Haven ratios are between 1.4 and 1.6. Supplementary Table 1 lists also the melting point temperature of the different compounds.

Supplementary Table 1: Diffusion coefficients $D$ ($10^{-5}$ cm$^2$ s$^{-1}$), densities $\rho$ (kg m$^{-3}$) and conductivities $\sigma$ (S cm$^{-1}$) of the different electrolytes at 1200 K and 773 K. Investigated salts: LiF, LiCl, LiI, LiF-LiCl$_{eut}$, LiF-LiI$_{eut}$, LiCl-LiI$_{eut}$, LiF-LiCl-LiI$_{eut}$, LiF-LiCl-LiI$_{400}$ and LiF-LiCl-LiI$_{450}$. Method (M) for evaluating the diffusion coefficients: mean square displacement (msd) and velocity autocorrelation function (vac). Conductivities calculated using Nernst-Einstein (NE), Green-Kubo (GK), and Einstein-Helfand (EH) theory. Haven ratio defined as $H = \sigma_{\mathrm{NE}}/\sigma_{\mathrm{GK}}$. The deviation of the real conductivity from the NE conductivity $\Delta$, defined as $\sigma = \sigma_{\mathrm{NE}}$ (1 - $\Delta$);[8] as real conductivity we used the GK conductivity.

| Electrolyte | $T_{\mathrm{m}}$ / K | M | $D_{\mathrm{Li^+}}$ | $D_{\mathrm{F^-}}$ | $D_{\mathrm{Cl^-}}$ | $D_{\mathrm{I^-}}$ | $\rho$ | $\rho_{\mathrm{exp}}$ | $\sigma_{\mathrm{NE}}$ | $\sigma_{\mathrm{GK}}$ | $\sigma_{\mathrm{EH}}$ | $\sigma_{\mathrm{exp}}$ | $H$ | $\Delta$ |
|---|---|---|---|---|---|---|---|---|---|---|---|---|---|---|
| 1200 K | | | | | | | | | | | | | | |
| LiF | 1121[9] | msd | 9.5 | 8.8 | | | 1958 | 1770[10] | 12.9 | 11.3 | 11.2 | 8.9[10] | 1.1 | 0.12 |
| | | vac | 9.1 | 8.1 | | | | | 12.2 | | | | 1.1 | 0.07 |
| LiCl | 883[9] | msd | 16.9 | | 11.7 | | 1369 | 1365[10] | 8.6 | 6.5 | 7.1 | 7.1[10] | 1.3 | 0.25 |
| | | vac | 16.1 | | 10.9 | | | | 8.1 | | | | 1.3 | 0.21 |
| LiI | 742[9] | msd | 21.0 | | | 10.8 | 2446 | 2689[10] | 5.4 | 3.5 | 3.4 | 5.1[10] | 1.5 | 0.35 |
| | | vac | 22.1 | | | 10.7 | | | 5.6 | | | | 1.6 | 0.37 |
| LiF-LiCl$_{eut}$ | 774,[11] 757[10] | msd | 14.3 | 10.4 | 11.2 | | 1451 | 1417[10] | 9.2 | 5.9 | 6.2 | 6.1[10] | 1.6 | 0.36 |
| | | vac | 13.6 | 10.5 | 11.1 | | | | 8.9 | | | | 1.5 | 0.34 |
| LiF-LiI$_{eut}$ | 684[11] | msd | 18.4 | 11.6 | | 11.2 | 2404 | 2647[12] | 5.7 | 3.6 | 3.9 | 4.0[12] | 1.6 | 0.37 |
| | | vac | 17.8 | 10.2 | | 10.9 | | | 5.5 | | | | 1.5 | 0.35 |
| LiCl-LiI$_{eut}$ | 641[11] | msd | 20.0 | | 12.1 | 11.4 | 2180 | 2340[12] | 6.3 | 4.6 | 4.8 | 6.1,[13] 5.4[12] | 1.4 | 0.28 |
| | | vac | 20.1 | | 11.5 | 11.2 | | | 6.2 | | | | 1.4 | 0.27 |
| LiF-LiCl-LiI$_{eut}$ | 614[11] | msd | 18.2 | 11.2 | 11.8 | 11.1 | 2177 | | 6.3 | 4.1 | 4.2 | 4.3[13] | 1.5 | 0.35 |
| | | vac | 17.8 | 10.6 | 12.4 | 10.7 | | | 6.2 | | | | 1.5 | 0.34 |
| LiF-LiCl-LiI$_{400}$ | $\approx$ 673[14] | msd | 16.5 | 10.9 | 11.7 | 11.0 | 2020 | | 6.9 | 4.5 | 4.5 | | 1.5 | 0.35 |
| | | vac | 16.5 | 9.6 | 12.0 | 11.5 | | | 6.9 | | | | 1.5 | 0.35 |
| LiF-LiCl-LiI$_{450}$ | $\approx$ 723[14] | msd | 15.5 | 10.5 | 11.4 | 10.5 | 1784 | | 7.8 | 4.8 | 5.2 | | 1.6 | 0.39 |
| | | vac | 15.1 | 10.4 | 10.7 | 11.6 | | | 7.6 | | | | 1.6 | 0.37 |
| 773 K | | | | | | | | | | | | | | |
| LiF-LiCl-LiI$_{eut}$ | | msd | 4.8 | 2.5 | 2.9 | 2.8 | 2628 | 2690[14] | 3.1 | 2.6 | 2.6 | 2.9[13] | 1.2 | 0.14 |
| | | vac | 4.8 | 2.5 | 2.7 | 2.8 | | | 3.0 | | | | 1.1 | 0.12 |
| LiF-LiCl-LiI$_{400}$ | | msd | 4.0 | 2.3 | 2.7 | 2.6 | 2421 | | 3.0 | 2.7 | 2.6 | | 1.1 | 0.10 |
| | | vac | 4.1 | 2.5 | 2.8 | 2.4 | | | 3.1 | | | | 1.1 | 0.12 |
| LiF-LiCl-LiI$_{450}$ | | msd | 3.5 | 2.2 | 2.5 | 2.3 | 2119 | | 3.2 | 2.7 | 2.5 | | 1.2 | 0.17 |
| | | vac | 3.6 | 2.1 | 2.6 | 2.4 | | | 3.3 | | | | 1.2 | 0.19 |

Supplementary Table 2: Diffusion coefficients $D$ ($10^{-5}$ cm$^2$ s$^{-1}$), densities $\rho$ (kg m$^{-3}$) and conductivity $\sigma$ (S cm$^{-1}$) of LiF-LiCl-LiI$_{eut}$ at different temperatures T (in K). Method (M) for the evaluation of the diffusion coefficients: mean square displacement (msd) and velocity autocorrelation function (vac). Conductivities calculated using Nernst-Einstein (NE), Green-Kubo (GK), and Einstein-Helfand (EH) theory. Experimental conductivity data taken from Masset et al.[13] measured between 623 - 873 K; the values are calculated using their equation. Haven ratio defined as $H = \sigma_{\mathrm{NE}}/\sigma_{\mathrm{GK}}$. The Nernst-Einstein deviation parameter $\Delta$ of the real conductivity from the NE conductivity, defined as $\sigma = \sigma_{\mathrm{NE}}\,(1 - \Delta)$;[8,15] as real conductivity we used the GK conductivity. The melting point of LiF-LiCl-LiI$_{eut}$ is 614 K.

| $T$ / K | M | $D_{\mathrm{Li^+}}$ | $D_{\mathrm{F^-}}$ | $D_{\mathrm{Cl^-}}$ | $D_{\mathrm{I^-}}$ | $\rho_{\mathrm{MD}}$ | $\rho_{\mathrm{exp}}$ | $\sigma_{\mathrm{NE}}$ | $\sigma_{\mathrm{GK}}$ | $\sigma_{\mathrm{EH}}$ | $\sigma_{\mathrm{exp}}$ | H | $\Delta$ |
|---|---|---|---|---|---|---|---|---|---|---|---|---|---|
| 623 | msd | 1.8 | 0.8 | 1.0 | 1.0 | 2809 | | 1.5 | 1.4 | 1.4 | 2.2 | 1.1 | 0.05 |
| | vac | 1.8 | 0.8 | 0.9 | 0.9 | | | 1.4 | | | | 1.0 | 0.03 |
| 723 | msd | 3.7 | 1.8 | 2.1 | 2.1 | 2688 | | 2.5 | 2.2 | 2.3 | 2.7 | 1.1 | 0.11 |
| | vac | 3.7 | 1.8 | 2.0 | 2.0 | | | 2.5 | | | | 1.1 | 0.10 |
| 773 | msd | 4.8 | 2.5 | 2.9 | 2.8 | 2628 | 2690[14] | 3.1 | 2.6 | 2.6 | 2.9 | 1.2 | 0.14 |
| | vac | 4.8 | 2.5 | 2.7 | 2.8 | | | 3.0 | | | | 1.1 | 0.12 |
| 1000 | msd | 11.2 | 6.6 | 6.9 | 6.8 | 2382 | | 5.1 | 3.7 | 3.3 | 3.7 | 1.4 | 0.27 |
| | vac | 11.0 | 6.9 | 6.8 | 6.8 | | | 5.0 | | | | 1.3 | 0.26 |
| 1200 | msd | 18.2 | 11.2 | 11.8 | 11.1 | 2177 | | 6.3 | 4.1 | 4.2 | 4.3 | 1.5 | 0.35 |
| | vac | 17.8 | 10.6 | 12.4 | 10.7 | | | 6.2 | | | | 1.5 | 0.34 |

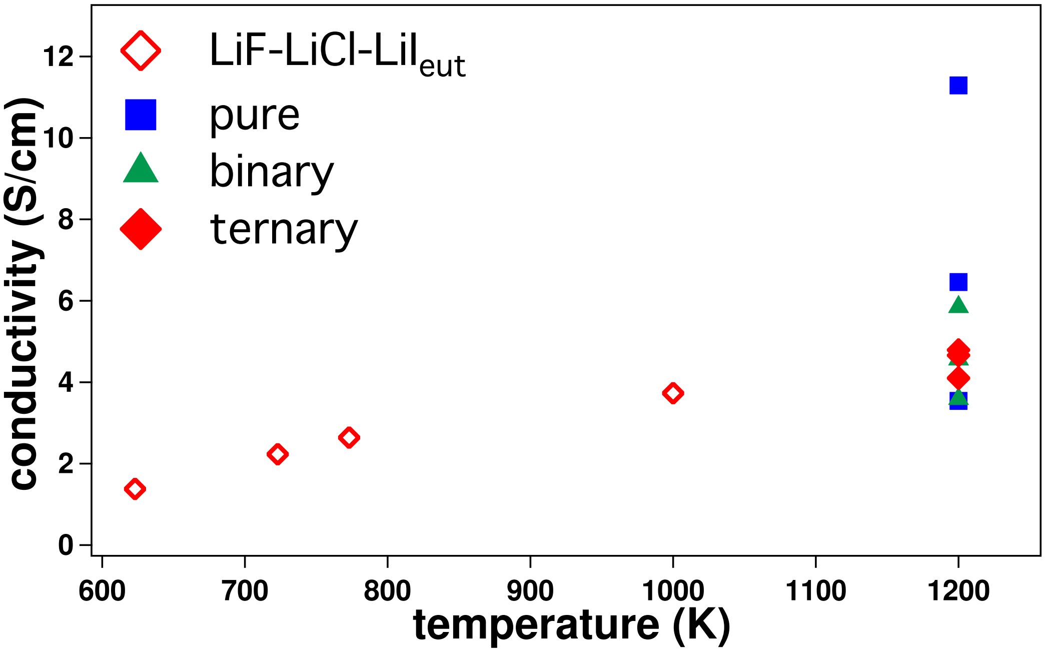


Supplementary Figure 2: GK conductivity vs. temperature.

# Supplementary Note 2

## Velocity autocorrelation functions

The velocity autocorrelation (vac) function reveals the underlying nature of the dynamical processes of a system. It reflects the forces that act on each particle (here ion), and how fast the velocity decorrelates with time. In a system with weak forces, the vac function is almost horizontal and simply exponentially decays. In a system with stronger forces, such as in a solid or liquid, the particles oscillates (reversing the velocity at the end of each oscillation) around positions where there is a balance between repulsive and attractive forces. In a solid, those position are well defined and the vac function will oscillate strongly from positive to negative values, but will still decay over time, as there are perturbative forces acting on the particles. In a liquid, diffusion causes those position to be not stable, and any oscillatory motion is rapidly destroyed. The vac function may show one very damped oscillation (a function with only one minimum) before decaying to zero. In simple terms this may be considered as a collision between two atoms before they rebound from one another and diffuse away.

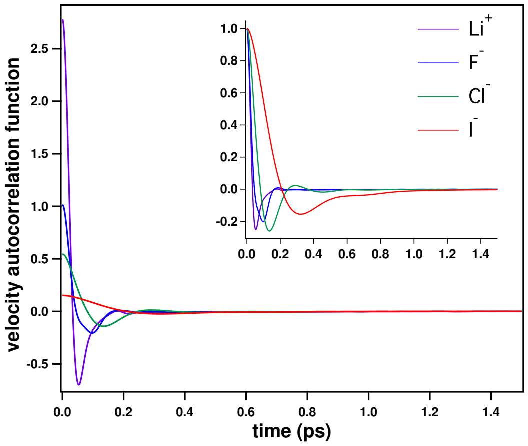


Supplementary Figure 3: Velocity autocorrelation function of LiF-LiCl-$LiI_{eut}$ at 773K. The inset shows the normalized vac function.

In Supplementary Fig. 3 the vac function for all ions in LiF-LiCl-$LiI_{eut}$ at 773 K is shown. In the inset that shows the normalized vac function, one can see that the decorrelation time correlates

with the mass of the ions. The velocity decorrelates fastest for $Li^+$ and decays slowest for $I^-$, meaning that the $Li^+$ is more affected by its surrounding than the heavier $I^-$. In Supplementary Fig. 4 a, the vac function of $Li^+$ is shown at 1200 K for the different investigated systems. It is clearly visible that the dynamics of $Li^+$ are a function of the composition. It is observed that $Li^+$ in molten LiF behaves most as if the liquid has well defined positions; this might be not surprising as the simulation temperature is only approx. 80 K above its melting point. In Supplementary Fig. 4 b, the vac function of $Li^+$ in LiF-LiCl-LiI$_{eut}$ is shown for the different temperatures. As expected the decay is getting faster the hotter the system gets.

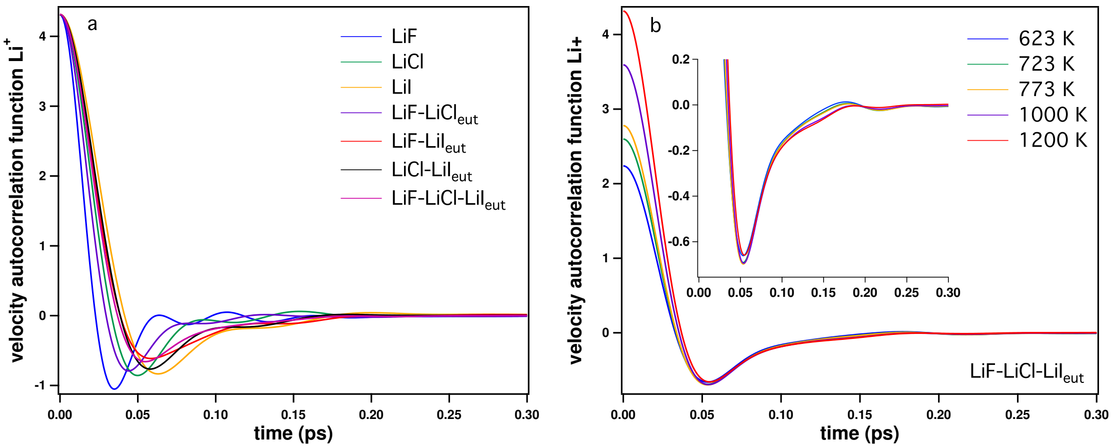


Supplementary Figure 4: Velocity autocorrelation function of $Li^+$ at 1200 K in various molten salts (a) and in LiF-LiCl-LiI$_{eut}$ at different temperatures (b).

## Current autocorrelation functions

In Supplementary Fig. 5 the current autocorrelation function (caf) and its running integral is shown for LiF-LiCl-LiI$_{eut}$ at different temperatures. The GK conductivity is evaluated from the running integral of the caf as the average value between 1 and 4 ps.

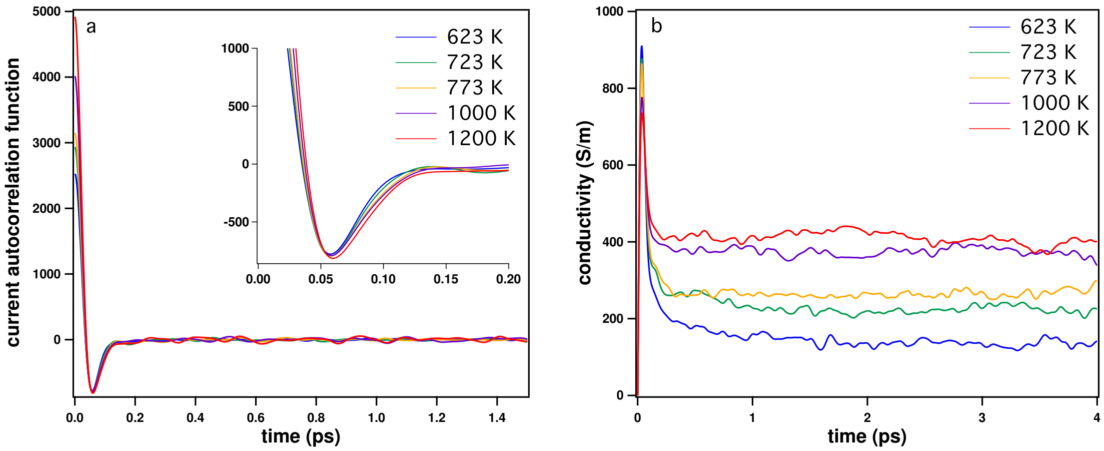


Supplementary Figure 5: Electric current autocorrelation function (a) and its running integral (b) representing the electrical conductivity of LiF-LiCl-LiI$_{eut}$ at different temperatures.

# Supplementary Note 3

## Plots showing diffusion coefficients and conductivity

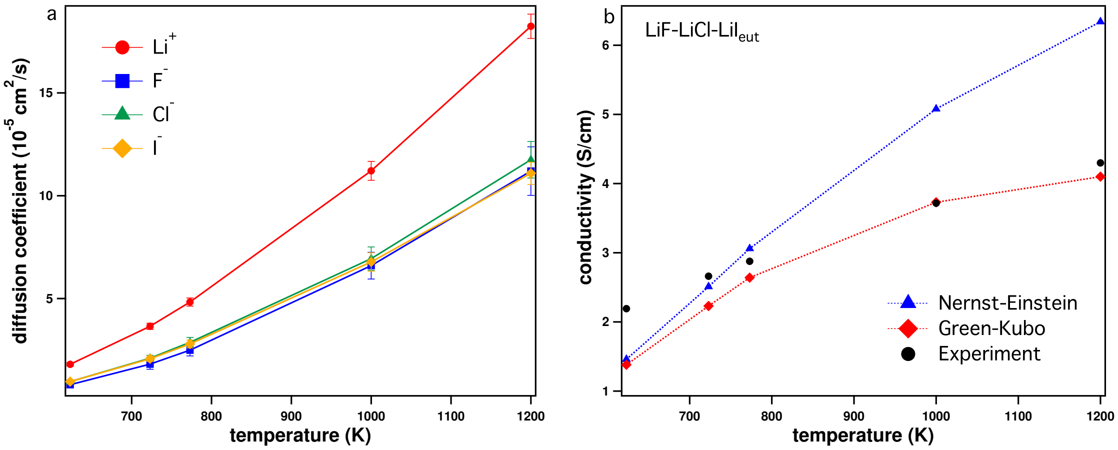


Supplementary Figure 6: (a) Diffusion coefficients (msd) and (b) conductivity of LiF-LiCl-LiI$_{eut}$ at different temperatures using Nernst-Einstein and Green-Kubo theory in comparison to experimental data.

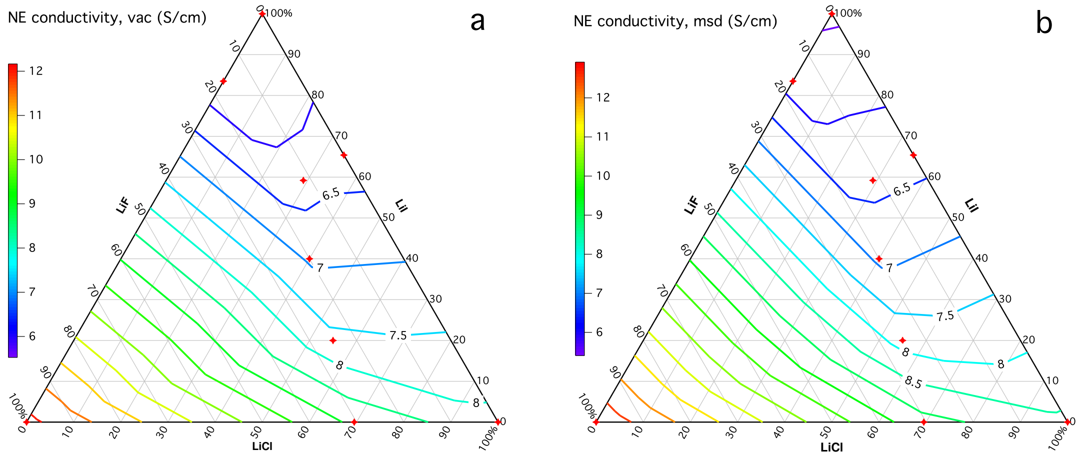


Supplementary Figure 7: Ternary diagram of the NE conductivity evaluated with (a) vac and (b) msd. Calculated points are shown as red diamonds; the contour lines are linearly interpolated.

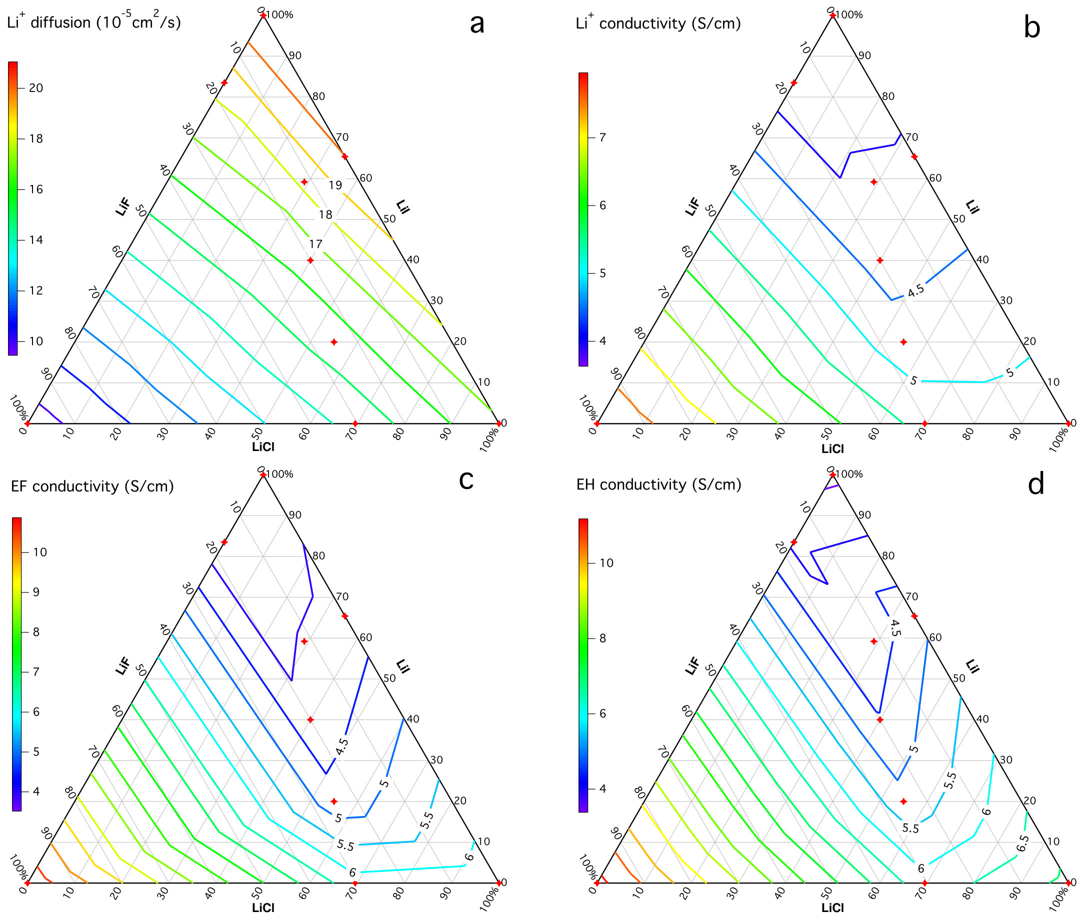


Supplementary Figure 8: Ternary diagram of (a) $Li^+$ diffusion coefficients in $10^{-5}$ $cm^2$ $s^{-1}$ (msd), (b) $Li^+$ conductivity in S $cm^{-1}$ (from EF simulation), (c) EF conductivity in S $cm^{-1}$ and (d) EH conductivity in S $cm^{-1}$ for salts containing LiF, LiCl, and/or LiI at 1200 K. Calculated points are shown as red diamonds; the contour lines are linearly interpolated.

# Supplementary Note 4

## Table listing mobilities and partial conductivities

The mobilities that were evaluated using linear regression show in general very high correlations, i.e. either +1.0 or -1.0, depending on the drift direction of the ions. However, we observed lower correlation coefficients for $Cl^-$ in certain mixtures where the $Cl^-$ mobilities seemed to be rather low (LiF-LiCl-$LiI_{eut}$ at 623 - 1000 K). For $Cl^-$ in LiF-LiCl-$LiI_{400}$ at 773 K, the correlation is extremely low with a mobility value of 0.1 x $10^{-8}$ $m^2$ $V^{-1}$ $s^{-1}$, i.e. almost zero. From the mobilities the partial conductivities were determined and it was checked whether they obey the universal

golden rule (see main article). In all cases the golden rule holds true. Therefore, the mobility values and the respective partial conductivity values seem to be reliable. The average error is estimated to be around 2 % for $Li^+$ and $I^-$, and 4 % for $F^-$. For $Cl^-$ also larger values are observed as its mobility and partial conductivity approach values close to zero.

Supplementary Table 3: Ion mobilities ($10^{-8}$ m$^2$ V$^{-1}$ s$^{-1}$), (partial) conductivities (S cm$^{-1}$) and Haven ratios of the different electrolytes at 1200 K and 773 K. Method (M) for evaluation of mobility $b$ and conductivity $\sigma$ values: (1) (EF, in bold) via non-equilibrium MD simulations applying an electric field or (2) (D) via equilibrium MD simulations using diffusion coefficients determined by the velocity autocorrelation function. The conductivities $\sigma_{\mathrm{EF}}$ and $\sigma_{\mathrm{NE}}$ are listed under $\sigma_{\mathrm{tot}}$, where $\sigma_{\mathrm{EF}}$ is calculated using the mobility values from the EF simulations and $\sigma_{\mathrm{NE}}$ using the mobility values determined from $D_{\mathrm{vac}}$. The Haven ratio is here defined as $H = \sigma_{\mathrm{NE}}/\sigma_{\mathrm{EF}}$.

| Electrolyte | M | $b_{\mathrm{Li}^+}$ | $b_{\mathrm{F}^-}$ | $b_{\mathrm{Cl}^-}$ | $b_{\mathrm{I}^-}$ | $\sigma_{\mathrm{Li}^+}$ | $\sigma_{\mathrm{F}^-}$ | $\sigma_{\mathrm{Cl}^-}$ | $\sigma_{\mathrm{I}^-}$ | $\sigma_{\mathrm{tot}}$ | $H$ | $\sigma_{\mathrm{GK}}$ |
|---|---|---|---|---|---|---|---|---|---|---|---|---|
| 1200 K | | | | | | | | | | | | |
| LiF | EF | **10.9** | **-4.0** | | | **8.0** | **2.9** | | | **10.9** | 1.1 | 11.3 |
| | D | 8.8 | -7.9 | | | 6.4 | 5.7 | | | 12.2 | | |
| LiCl | EF | **17.0** | | **-3.3** | | **5.3** | | **1.0** | | **6.3** | 1.3 | 6.5 |
| | D | 15.5 | | -10.6 | | 4.8 | | 3.3 | | 8.1 | | |
| LiI | EF | **20.6** | | | **-1.1** | **3.6** | | | **0.2** | **3.8** | 1.5 | 3.5 |
| | D | 21.4 | | | -10.3 | 3.8 | | | 1.8 | 5.6 | | |
| LiF-LiCl$_{eut}$ | EF | **14.2** | **3.5** | **-4.8** | | **5.3** | **-0.4** | **1.3** | | **6.2** | 1.4 | 5.9 |
| | D | 13.2 | -10.1 | -10.7 | | 4.9 | 1.2 | 2.8 | | 8.9 | | |
| LiF-LiI$_{eut}$ | EF | **18.2** | **10.9** | | **-1.5** | **3.6** | **-0.4** | | **0.3** | **3.5** | 1.6 | 3.6 |
| | D | 17.3 | -9.9 | | -10.6 | 3.5 | 0.3 | | 1.8 | 5.5 | | |
| LiCl-LiI$_{eut}$ | EF | **19.8** | | **2.4** | **-2.0** | **4.1** | | **-0.2** | **0.3** | **4.2** | 1.5 | 4.6 |
| | D | 19.4 | | -11.1 | -10.8 | 4.0 | | 0.8 | 1.5 | 6.2 | | |
| LiF-LiCl-LiI$_{eut}$ | EF | **18.7** | **10.6** | **2.0** | **-2.3** | **4.1** | **-0.3** | **-0.1** | **0.3** | **4.0** | 1.5 | 4.1 |
| | D | 17.3 | -10.3 | -12.0 | -10.3 | 3.8 | 0.3 | 0.8 | 1.4 | 6.2 | | |
| LiF-LiCl-LiI$_{400}$ | EF | **16.7** | **8.5** | **0.5** | **-3.1** | **4.3** | **-0.4** | **-0.05** | **0.3** | **4.1** | 1.7 | 4.5 |
| | D | 16.0 | -9.3 | -11.6 | -11.2 | 4.1 | 0.5 | 1.2 | 1.1 | 6.9 | | |
| LiF-LiCl-LiI$_{450}$ | EF | **15.5** | **7.1** | **-1.6** | **-4.4** | **4.7** | **-0.5** | **0.3** | **0.3** | **4.7** | 1.6 | 4.8 |
| | D | 14.6 | -10.0 | -10.4 | -11.2 | 4.4 | 0.8 | 1.7 | 0.7 | 7.6 | | |
| 773 K | | | | | | | | | | | | |
| LiF-LiCl-LiI$_{eut}$ | EF | **9.9** | **5.3** | **0.4** | **-1.1** | **2.7** | **-0.2** | **-0.03** | **0.2** | **2.6** | 1.1 | 2.6 |
| | D | 7.1 | -3.7 | -4.1 | -4.1 | 1.9 | 0.1 | 0.3 | 0.7 | 3.0 | | |
| LiF-LiCl-LiI$_{400}$ | EF | **8.5** | **4.3** | **0.1** | **-1.5** | **2.6** | **-0.3** | **-0.01** | **0.2** | **2.5** | 1.2 | 2.7 |
| | D | 6.1 | -3.7 | -4.2 | -3.6 | 1.9 | 0.2 | 0.5 | 0.4 | 3.1 | | |
| LiF-LiCl-LiI$_{450}$ | EF | **7.7** | **3.2** | **-0.9** | **-2.0** | **2.8** | **-0.3** | **0.2** | **0.1** | **2.8** | 1.2 | 2.7 |
| | D | 5.5 | -3.1 | -3.9 | -3.6 | 2.0 | 0.3 | 0.8 | 0.3 | 3.3 | | |

With the following parametric expression the conductivity data can be approximated in dependence of the salt's composition ($x_i$ being the molar fraction) and its temperature $T$:

$$ln(\sigma_{x_i,T}) = \alpha - \frac{\beta}{T} + \frac{\gamma}{x_{\mathrm{LiF}}\delta + x_{\mathrm{LiCl}}\epsilon + x_{\mathrm{LiI}}\zeta} + \frac{\eta}{(x_{\mathrm{LiF}}\delta + x_{\mathrm{LiCl}}\epsilon + x_{\mathrm{LiI}}\zeta)^2} \quad (7)$$

with $\alpha$ = 2.145, $\beta$ = 1048.61, $\gamma$ = 655.73, $\delta$ = 592.51, $\epsilon$ = 1290.25, $\zeta$ = 8294.43 and $\eta$ = 1379.22.

Supplementary Table 4: Comparison of the conductivity values determined from the electric field simulations vs. conductivity values (in S cm$^{-1}$) calculated using the parametric expression.

| salt | $T$ / K | $x_{\mathrm{LiF}}$- $x_{\mathrm{LiCl}}$-$x_{\mathrm{LiI}}$ | $\sigma_{\mathrm{EF}}$ | $\sigma_{\mathrm{calc}}$ |
|---|---|---|---|---|
| LiF | 1200 | 1 - 0 - 0 | 10.86 | 10.82 |
| LiCl | 1200 | 0 - 1 - 0 | 6.35 | 5.93 |
| LiI | 1200 | 0 - 0 - 1 | 3.84 | 3.86 |
| LiF-LiCl$_{eut}$ | 1200 | 0.305 - 0.695 - 0 | 6.19 | 6.56 |
| LiF-LiI$_{eut}$ | 1200 | 0.165 - 0 - 0.835 | 3.53 | 3.91 |
| LiCl-LiI$_{eut}$ | 1200 | 0 - 0.346 - 0.654 | 4.17 | 3.99 |
| LiF-LiCl-LiI$_{eut}$ | 1200 | 0.117 - 0.291 - 0.592 | 4.05 | 4.03 |
| LiF-LiCl-LiI$_{400}$ | 1200 | 0.2 - 0.4 - 0.4 | 4.13 | 4.21 |
| LiF-LiCl-LiI$_{450}$ | 1200 | 0.25 - 0.55 - 0.2 | 4.69 | 4.63 |
| LiF-LiCl-LiI$_{eut}$ | 773 | 0.117 - 0.291 - 0.592 | 2.63 | 2.49 |
| LiF-LiCl-LiI$_{400}$ | 773 | 0.2 - 0.4 - 0.4 | 2.54 | 2.60 |
| LiF-LiCl-LiI$_{450}$ | 773 | 0.25 - 0.55 - 0.2 | 2.81 | 2.86 |
| LiF-LiCl-LiI$_{eut}$ | 623 | 0.117 - 0.291 - 0.592 | 1.66 | 1.79 |
| LiF-LiCl-LiI$_{eut}$ | 723 | 0.117 - 0.291 - 0.592 | 2.17 | 2.26 |
| LiF-LiCl-LiI$_{eut}$ | 1000 | 0.117 - 0.291 - 0.592 | 3.61 | 3.38 |

## Transference numbers

Transference numbers are calculated from the partial conductivities determined from the non-equilibrium simulations where an external electric field was applied, with $t_i = \sigma_i/\sigma_{\mathrm{tot}}$. For comparison also the usually calculated transference numbers $t_i^{\mathrm{NE}}$ using the Nernst-Einstein partial conductivities are given. It is very clear that $t_i^{\mathrm{NE}}$ are very different from the accurate transference numbers, both quantitatively and qualitatively, and thus misleading. By definition the sum over all $t_i$ values is equal to 1.

Supplementary Table 5: Transference numbers for LiF-LiCl-LiI$_{eut}$ at different temperatures.

| $T$ / K | $t_{\mathrm{Li^+}}$ | $t_{\mathrm{F^-}}$ | $t_{\mathrm{Cl^-}}$ | $t_{\mathrm{I^-}}$ | $t_{\mathrm{Li^+}}^{\mathrm{NE}}$ | $t_{\mathrm{F^-}}^{\mathrm{NE}}$ | $t_{\mathrm{Cl^-}}^{\mathrm{NE}}$ | $t_{\mathrm{I^-}}^{\mathrm{NE}}$ |
|---|---|---|---|---|---|---|---|---|
| 623 | 1.00 | -0.06 | -0.01 | 0.07 | 0.67 | 0.03 | 0.10 | 0.20 |
| 723 | 1.01 | -0.07 | -0.02 | 0.07 | 0.65 | 0.04 | 0.10 | 0.21 |
| 773 | 1.01 | -0.06 | -0.01 | 0.07 | 0.64 | 0.04 | 0.11 | 0.22 |
| 1000 | 1.01 | -0.06 | -0.02 | 0.07 | 0.62 | 0.05 | 0.11 | 0.23 |
| 1200 | 1.03 | -0.07 | -0.03 | 0.08 | 0.61 | 0.04 | 0.12 | 0.22 |

Supplementary Table 6: Transference numbers for different electrolytes at 1200 K and 773 K.

| Electrolyte | $t_{\mathrm{Li^+}}$ | $t_{\mathrm{F^-}}$ | $t_{\mathrm{Cl^-}}$ | $t_{\mathrm{I^-}}$ | $t_{\mathrm{Li^+}}^{\mathrm{NE}}$ | $t_{\mathrm{F^-}}^{\mathrm{NE}}$ | $t_{\mathrm{Cl^-}}^{\mathrm{NE}}$ | $t_{\mathrm{I^-}}^{\mathrm{NE}}$ |
|---|---|---|---|---|---|---|---|---|
| 1200 K | | | | | | | | |
| LiF | 0.73 | 0.27 | | | 0.53 | 0.47 | | |
| LiCl | 0.84 | | 0.16 | | 0.60 | | 0.40 | |
| LiI | 0.95 | | | 0.05 | 0.67 | | | 0.33 |
| LiF-LiCl$_{eut}$ | 0.86 | -0.06 | 0.20 | | 0.56 | 0.13 | 0.31 | |
| LiF-LiI$_{eut}$ | 1.03 | -0.10 | | 0.07 | 0.62 | 0.06 | | 0.32 |
| LiCl-LiI$_{eut}$ | 0.98 | | -0.04 | 0.06 | 0.64 | | 0.13 | 0.23 |
| LiF-LiCl-LiI$_{eut}$ | 1.03 | -0.07 | -0.03 | 0.08 | 0.61 | 0.04 | 0.12 | 0.22 |
| LiF-LiCl-LiI$_{400}$ | 1.04 | -0.11 | -0.01 | 0.08 | 0.59 | 0.07 | 0.17 | 0.17 |
| LiF-LiCl-LiI$_{450}$ | 1.00 | -0.12 | 0.06 | 0.06 | 0.58 | 0.10 | 0.23 | 0.09 |
| 773 K | | | | | | | | |
| LiF-LiCl-LiI$_{eut}$ | 1.01 | -0.06 | -0.01 | 0.07 | 0.64 | 0.04 | 0.11 | 0.22 |
| LiF-LiCl-LiI$_{400}$ | 1.04 | -0.11 | -0.003 | 0.07 | 0.61 | 0.07 | 0.17 | 0.15 |
| LiF-LiCl-LiI$_{450}$ | 0.99 | -0.10 | 0.06 | 0.05 | 0.60 | 0.09 | 0.24 | 0.08 |

# Supplementary Note 5

## Radial distribution functions

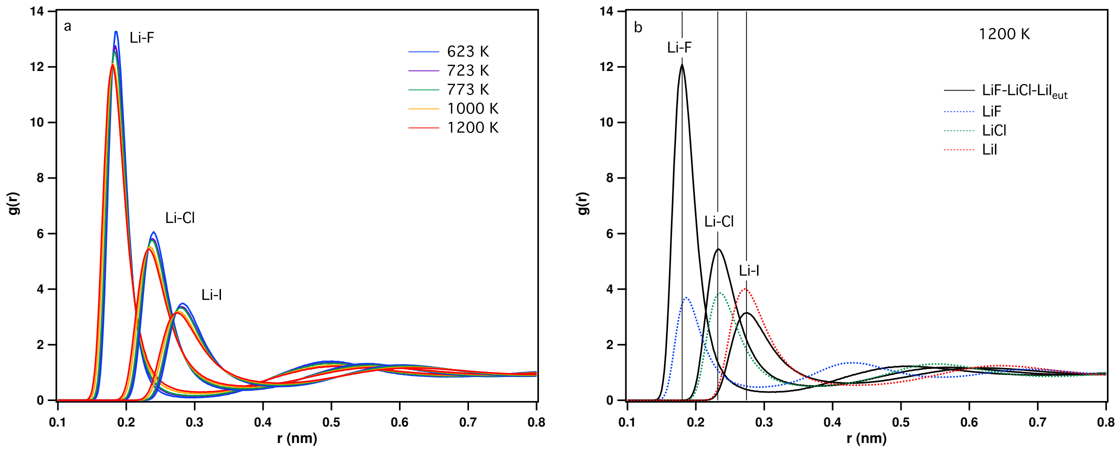


Supplementary Figure 9: Radial distribution functions for LiF-LiCl-LiI$_{eut}$ at different temperatures (a) and for LiF-LiCl-LiI$_{eut}$, LiF, LiCl and LiI at 1200 K (b).

# Supplementary Note 6

## Evaluation of bond distances, coordination numbers, lifetimes and Gibbs free energy of activation of bond breaking

Dynamics can be studied quantitatively through the Luzar-Chandler analysis that was developed for hydrogen bonds in liquid water,[16,17] but that has previously been applied to liquid mixtures[18] as well as protein-ion interactions.[19] This method yields lifetimes for, in this case, the breaking of ion-pair contacts in a melt, as we have investigated earlier.[20] Based upon the Eyring equation, the Gibbs free energy of activation $\Delta^{\ddagger}\mathrm{G}$ for breaking an ion contact can be calculated from the lifetime $\tau$[18] under the assumption that the transmission coefficient $\kappa$ is equal to one:

$$\Delta^{\ddagger}\mathrm{G} = RT\ln\frac{\kappa k_\mathrm{B}T\tau}{h}, \tag{8}$$

where $k_\mathrm{B}$ is the Boltzmann's constant, $T$ is the temperature and $h$ is the Planck's constant. For the Luzar-Chandler analysis a cut-off has to be selected at which the ion pair is not considered as an ion pair anymore; this was evaluated from the radial distribution function and selected to be the minimum after the first peak position, here defined as the maximum ion-pair bond length. From the radial distribution function (rdf) also a cumulative coordination number can be calculated; the given coordination numbers were evaluated from the cumulative radial distribution function at the distance of first minimum in the rdf that was also used for the Luzar-Chandler analysis.

Supplementary Table 7: Lifetimes $\tau$ (ps), Gibbs free energies of activation $\Delta^{\ddagger}$G (kJ mol$^{-1}$) of bond breaking, coordination numbers $N$ and interionic distances $r$ (pm) for the ion pairs in the pure, binary and ternary salt melts at 1200 K. The coordination numbers for the binary and ternary mixtures in the table reflect the average number of Li-ions around one anion.

| | $\tau$ | | | $\Delta^{\ddagger}$G | | | $N$ | | | $r$ | | |
|---|---|---|---|---|---|---|---|---|---|---|---|---|
| Salt \ ion pair | LiF | LiCl | LiI | LiF | LiCl | LiI | LiF | LiCl | LiI | LiF | LiCl | LiI |
| LiF | 1.5 | | | 36.3 | | | 5.1 | | | 186 | | |
| LiCl | | 1.7 | | | 37.6 | | | 4.8 | | | 236 | |
| LiI | | | 2.2 | | | 40.2 | | | 4.4 | | | 272 |
| LiF-LiCl$_{eut}$ | 2.6 | 1.7 | | 41.5 | 37.4 | | 4.0 | 5.2 | | 182 | 238 | |
| LiF-LiI$_{eut}$ | 4.9 | | 2.2 | 48.0 | | 40.1 | 3.5 | | 4.6 | 178 | | 272 |
| LiCl-LiI$_{eut}$ | | 2.5 | 2.0 | | 41.1 | 39.2 | | 4.1 | 4.7 | | 232 | 272 |
| LiF-LiCl-LiI$_{eut}$ | 4.3 | 2.4 | 2.0 | 46.6 | 40.9 | 39.1 | 3.5 | 4.3 | 4.8 | 180 | 232 | 276 |
| LiF-LiCl-LiI$_{400}$ | 3.6 | 2.2 | 1.9 | 45.0 | 39.9 | 38.4 | 3.7 | 4.5 | 5.1 | 182 | 234 | 276 |
| LiF-LiCl-LiI$_{450}$ | 3.1 | 1.9 | 1.7 | 43.3 | 38.6 | 37.3 | 3.8 | 4.7 | 5.4 | 180 | 236 | 278 |

# Supplementary Note 7

## Experimental melting points and conductivity values

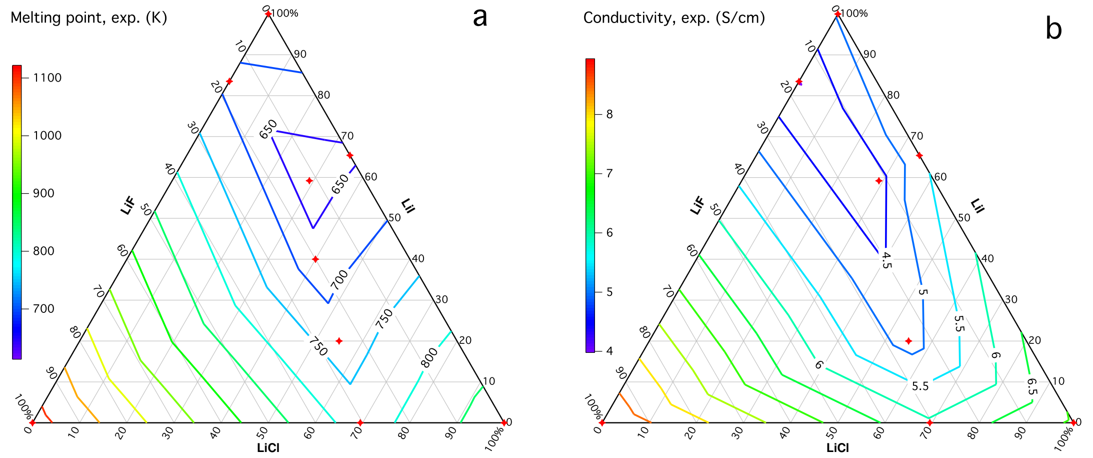


Supplementary Figure 10: Ternary diagram of (a) experimental melting points and (b) experimental conductivity at 1200 K (besides LiF-LiCl-LiI$_{400\ and\ 450}$ that are the calculated GK data points). The contour lines are linearly interpolated.

# Supplementary References


(1) Morgan, B.; Madden, P. A. Ion mobilities and microscopic dynamics in liquid (Li, K) Cl. *J. Chem. Phys.* **2004**, *120*, 1402–1413.

(2) Dommert, F.; Schmidt, J.; Qiao, B.; Zhao, Y.; Krekeler, C.; Delle Site, L.; Berger, R.; Holm, C. A comparative study of two classical force fields on statics and dynamics of [EMIM][$BF_4$] investigated via molecular dynamics simulations. *J. Chem. Phys.* **2008**, *129*, 224501.

(3) Michalowsky, J.; Zeman, J.; Holm, C.; Smiatek, J. A polarizable MARTINI model for monovalent ions in aqueous solution. *J. Chem. Phys.* **2018**, *149*, 163319.

(4) Allen, M. P.; Tildesley, D. J. *Computer Simulation of Liquids*; Oxford Science Publications: Oxford, 1987.

(5) Kowsari, M. H.; Fakhraee, M. Influence of butyl side chain elimination, tail amine functional addition, and C2 methylation on the dynamics and transport properties of imidazolium-based [$Tf2N^-$] ionic liquids from molecular dynamics simulations. *J. Chem. Eng. Data* **2015**, *60*, 551–560.

(6) Schröder, C.; Haberler, M.; Steinhauser, O. On the computation and contribution of conductivity in molecular ionic liquids. *J. Chem. Phys.* **2008**, *128*, 134501.

(7) Dommert, F.; Holm, C. Refining classical force fields for ionic liquids: theory and application to [MMIM][Cl]. *Phys. Chem. Chem. Phys.* **2013**, *15*, 2037–2049.

(8) Harris, K. R. Comment on "Ionic Conductivity, Diffusion Coefficients, and Degree of Dissociation in Lithium Electrolytes, Ionic Liquids, and Hydrogel Polyelectrolytes". *J. Phys. Chem. B* **2018**, *122*, 10964–10967.

(9) Sirdeshmukh, D. B.; Sirdeshmukh, L.; Subhadra, K. G. *Alkali Halides*, 1st ed.; Springer: Berlin, Heidelberg, New York, 2001.

(10) Janz, G. J.; Allen, C. B.; Bansal, N.; Murphy, R.; Tomkins, R. *Physical properties data compilations relevant to energy storage. II. Molten salts: data on single and multi-component salt systems*; National Standard Reference Data System, 1979.

(11) Masset, P.; Guidotti, R. A. Thermal activated (thermal) battery technology: Part II. Molten salt electrolytes. *J. Power Sources* **2007**, *164*, 397–414.

(12) Janz, G.; Tomkins, R.; Allen, C. Molten salts: Volume 4, part 4 mixed halide melts electrical conductance, density, viscosity, and surface tension data. *J. Phys.Chem. Ref. Data* **1979**, *8*, 125–302.

(13) Masset, P.; Henry, A.; Poinso, J.-Y.; Poignet, J.-C. Ionic conductivity measurements of molten iodide-based electrolytes. *J. Power Sources* **2006**, *160*, 752–757.

(14) Masset, P. Iodide-based electrolytes: A promising alternative for thermal batteries. *J. Power Sources* **2006**, *160*, 688–697.

(15) Harris, K. R. Can the transport properties of molten salts and ionic liquids be used to determine ion association? *J. Phys. Chem. B* **2016**, *120*, 12135–12147.

(16) Luzar, A.; Chandler, D. Effect of environment on hydrogen bond dynamics in liquid water. *Phys. Rev. Lett.* **1996**, *76*, 928–931.

(17) Luzar, A.; Chandler, D. Hydrogen-bond kinetics in liquid water. *Nature* **1996**, *379*, 55–57.

(18) van der Spoel, D.; van Maaren, P. J.; Larsson, P.; Tîmneanu, N. Thermodynamics of hydrogen bonding in hydrophilic and hydrophobic media. *J. Phys. Chem. B.* **2006**, *110*, 4393–4398.

(19) Larsson, D. S. D.; van der Spoel, D. Screening for the location of rna using the chloride ion distribution in simulations of virus capsids. *J. Chem. Theory Comput.* **2012**, *8*, 2474–2483.

(20) Walz, M.-M.; van der Spoel, D. Molten alkali halides - temperature dependence of structure, dynamics and thermodynamics. *Phys. Chem. Chem. Phys.* **2019**, *21*, 8516–18524, DOI: 10.1039/C9CP03603B.
