# Supplementary material for: Microscopic origins of conductivity in molten salts unraveled by computer simulations
Source: Commun Chem. 2021 Jan 27;4:9. doi: 10.1038/s42004-020-00446-2 (PMC9814786; doi:10.1038/s42004-020-00446-2)
Supplement: Supplementary file 3 — Description of Additional Supplementary Files [file 42004_2020_446_MOESM3_ESM.pdf]

Description of Additional Supplementary Files

File name: Supplementary Movie 1

Description: Molten LiF-LiCl-LiIeut at 1200 K with an applied electric field (0.4 V/nm), 1 ns.

File name: Supplementary Movie 2

Description: Molten LiF-LiCl-LiIeut at 1200 K with an applied electric field (0.4 V/nm), 50 ps. The coordination sphere is indicated for the shown ions.
